# Molecular characterisation and morphological description of two new species of *Hepatozoon* Miller, 1908 (Apicomplexa: Adeleorina: Hepatozoidae) infecting leukocytes of African leopards *Panthera pardus pardus* (L.)

**DOI:** 10.1186/s13071-020-3933-6

**Published:** 2020-05-01

**Authors:** Michelle van As, Edward C. Netherlands, Nico J. Smit

**Affiliations:** 1 Department of Zoology and Entomology, University of the Free State, Qwaqwa campus, Private Bag X13, Phuthaditjhaba, 9866 South Africa; 2 Water Research Group, Unit for Environmental Sciences and Management, North-West University, Private Bag X6001, Potchefstroom, 2520 South Africa

**Keywords:** *Hepatozoon*, African leopard, Feline haemogregarines, Haemoparasites

## Abstract

**Background:**

The African leopard *Panthera pardus pardus* (L.) is currently listed as a vulnerable species on the IUCN (International Union for the Conservation of Nature) red list of threatened species due to ongoing population declines. This implies that leopard-specific parasites are also vulnerable to extinction. Intracellular apicomplexan haemoparasites from the genus *Hepatozoon* Miller, 1908 have been widely reported from wild carnivores in Africa, including non-specific reports from leopards. This paper describes two new haemogregarines in captive and wild leopards from South Africa and provides a tabular summary of these species in relation to species of *Hepatozoon* reported from mammalian carnivores.

**Methods:**

Blood was collected from nine captive and eight wild leopards at various localities throughout South Africa. Thin blood smears were Giemsa-stained and screened for intraleukocytic haemoparasites. Gamont stages were micrographed and morphometrically compared with existing literature pertaining to infections in felid hosts. Haemogregarine specific primer set 4558F and 2733R was used to target the *18S* rRNA gene for molecular analysis. Resulting sequences were compared to each other and with other available representative mammalian carnivore *Hepatozoon* sequences from GenBank.

**Results:**

Two species of *Hepatozoon* were found in captive and wild leopards. Of the 17 leopards screened, eight were infected with one or both morphologically and genetically distinct haemogregarines. When compared with other species of *Hepatozoon* reported from felids, the two species from this study were morphometrically and molecularly distinct. Species of *Hepatozoon* from this study were observed to exclusively parasitize a particular type of leukocyte, with *Hepatozoon luiperdjie* n. sp. infecting neutrophils and *Hepatozoon ingwe* n. sp. infecting lymphocytes. Phylogenetic analysis showed that these haemogregarines are genetically distinct, with *Hepatozoon luiperdjie* n. sp. and *Hepatozoon ingwe* n. sp. falling in well supported separate clades.

**Conclusions:**

To our knowledge, this is the first morphometric and molecular description of *Hepatozoon* in captive and wild African leopards in South Africa. This study highlights the value of using both morphometric and molecular characteristics when describing species of *Hepatozoon* from felid hosts.

## Background

Members of the genus *Hepatozoon* Miller, 1908 are intracellular apicomplexan haemogregarines (Apicomplexa Levine, 1970: Adeleorina Léger, 1911: Hepatozoidae Wenyon, 1926) widely reported from amphibians, reptiles, birds and mammals, specifically including carnivores such as wild felids [1]. Feline hepatozoonosis was first reported in the early 1900’s by Patton [2], who described an intraleukocytic parasite *Leucocytozoon felis domestici* Patton, 1908 from an domestic cat in India. Eighteen years later, Wenyon [3] distinguished a capsule encasing this parasite that was overlooked by Patton [2], and then reclassified this parasite as a species of *Hepatozoon*, apparently morphologically identical to those found in hyenas, dogs and jackals [2, 3]. In the late 1990’s Beaufils et al. [4] proposed that canid and felid *Hepatozoon* are separate species, due to their morphological differences. The majority of species of *Hepatozoon* detected in African carnivores were previously identified as *Hepatozoon canis* (James, 1905), until Levine [5] suggested that host species should be taken into account when determining the identity of these parasites. Peirce et al. [6] supported this by suggesting that researchers are wrong to assume that all species of *Hepatozoon* from African carnivore are synonyms of *H. canis.*

From a parasitological perspective, the leopard *Panthera pardus* (L.) is one of the least studied big cats. Known protozoan parasites in leopards include species of *Toxoplasma* (Nicolle & Manceaux, 1908), *Sarcocystis* Lankester, 1882, *Hepatozoon*, *Giardia* Künstler, 1882 and *Isospora* Schneider, 1881 [7]. Brocklesby & Vidler [8] were the first to report *Hepatozoon*-like organisms from a free-ranging African leopard *P. pardus pardus* (Linnaeus 1758) in Kenya. In the early 1970’s, Keymer [9] reported *Hepatozoon canis*-like schizonts from the cardiac muscle of a leopard in Central Africa and four years later McCully et al. [10] reported *Hepatozoon*-like parasites from leopards in South Africa’s KwaZulu-Natal Province. More recently, Pawar et al. [11] and Khoshnegah et al. [12] detected what they thought to be *Hepatozoon felis* (Patton, 1908) from two free-ranging Indian leopards *Panthera pardus fusca* (Meyer) as well as an unnamed species of *Hepatozoon* from a single free-ranging Persian leopard *Panthera pardus ciscausica* (Satunin).

Prior to the 21st century, classification of species of *Hepatozoon* was based on their life history, host identity and morphological characteristics. However, with recent advances in molecular techniques, phylogenetic analyses on the relationships between species have become possible [13–16]. Morphological characteristics used to distinguish between species of *Hepatozoon* include gamont and nucleus dimensions; position of the nucleus within the gamont; number and arrangement of vacuoles and staining properties [17, 18], as well as characteristics of other developmental stages [19, 20]. In Africa, domestic dogs and wild carnivores have been reported to have cases of asymptomatic hepatozoonosis, caused mostly by *H. canis* and *H. felis* [6, 10, 21–25]. Although, *H. canis* and *H. felis* are not specific to either canids or felids, they have been reported infecting both carnivore families [26–28].

Due to constant improvement of molecular techniques, the number of studies on haemogregarines has systematically increased, with several studies relying solely on these methods to detect species of *Hepatozoon* in their hosts [11, 28–32]. Recent research has shown that a difference in p-distance of between 1–2% of the *18S* rRNA gene is sufficient to distinguish between species of haemogregarines if supported by morphological data [16, 18, 33, 34]. Other studies, such as Metzger et al. [35], incorporate both molecular and some degree of morphological investigations, showing the importance of utilizing a more holistic approach when distinguishing among species of *Hepatozoon*.

The objectives of the present study were to (i) investigate whether captive and wild leopards in South Africa are infected with species of *Hepatozoon*; (ii) identify any infections found using both molecular analysis of a fragment of the *18S* rRNA gene and morphological characteristics of the gamont stage in peripheral blood; (iii) determine if any of the *Hepatozoon* spp. identified can be linked to hepatozoonosis based on clinical symptoms of the host. This study is the first report on the molecular and morphological characteristics of *Hepatozoon* species infecting captive and wild leopards in South Africa.

## Methods

### Study area, *Panthera pardus pardus* collection and blood preparation

Blood samples were obtained from nine captive and eight wild leopards in South Africa (Table 1), representative of three core wild populations as identified by Daly et al. [36]. All live leopards were sedated and sampled by a qualified veterinarian surgeon following standard procedures. Blood scabs were collected from the frozen carcass of an erythristic (red) female leopard in the Mpumalanga Province (25°9′51.31ʺS, 30°26′55.46ʺE). Captive-bred leopards were sampled at two facilities in Bloemfontein, Free State Province (29°4′9.32ʺS, 26°9′34.40ʺE) (29°6′52.28ʺS, 26°12′15.80ʺE), and at one facility in the Mpumalanga Province (24°30′52.44ʺS, 30°54′8.82ʺE). Wild leopards were sampled in the Mpumalanga (25°9′51.31ʺS, 30°26′55.46ʺE) (24°34′43.50ʺS, 31°25′46.48ʺE) and northern Limpopo provinces (23°02′17.1ʺS, 29°26′26.5ʺE). Data collected on leopards included: geographical location, weight, age, physical condition, diet, parasite treatment, possible injuries, physical measurements, and possible clinical symptoms.

**Table 1:** Leopards sampled during this study from January 2013 to December 2015

Individual code	Sex	Color variation	Age class	Location
Captive leopards				
CF1	Female	Regular	Adult	BFNZoo
CF2	Female	Regular	Adult	CHXP
CF3	Female	Melanistic	Subadult	CHXP
CF4	Female	Melanistic	Adult	CHXP
CF5	Female	Regular	Adult	MOH
CM1	Male	Regular	Subadult	CHXP
CM2	Male	Melanistic	Adult	CHXP
CM3	Male	Regular	Adult	CHXP
CM4	Male	Regular	Subadult	MOH
Wild leopards				
WF1	Female	Regular	Adult	LA
WF2	Female	Erythristic	Adult	LA
WF3	Female	Regular	Subadult	GKCA
WM1	Male	Regular	Adult	LRC
WM2	Male	Regular	Adult	LRC
WM3	Male	Regular	Adult	LRC
WM4	Male	Regular	Adult	GKCA
WM5	Male	Regular	Adult	GKCA

*Abbreviations*: BFNZoo, Bloemfontein Zoo; CHXP, Cheetah Experience; GKCA, Greater Kruger Conservation Area; LA, Lydenburg surrounding area; LRC, Lajuma Research Centre; MOH, Moholoholo Wildlife Rehabilitation Centre

For this study, leopards are classified as ‘captive’ if they were born or raised in captivity from an early age (< 1-month-old). Leopards are classified as ‘wild’ when born or caught at an adult stage in the wild or when held at rehabilitation centres for less than six months to be relocated. Age classes were allocated to all leopards sampled following Fattebert et al. [37], with cubs (< 1 year), subadults (1–3 years) and adults (> 3 years). Wild leopards were aged according to the nature of tooth wear and a combination of morphological cues as described by Stander [38] and Balme et al. [39] respectively. Captive leopards were aged according to the information provided by their *ex situ* managers.

Prior to blood collection, the dense pelt over the identified area of collection was shaved with a clipper and wiped with an alcohol-soaked cotton ball, which helped remove external skin contaminants and improved visualisation of the vein. Peripheral blood was usually collected from the jugular or the cephalic vein by venipuncture with the use of a sterile Vacutainer system, in BD Vacutainer® (Franklin Lakes, USA) CAT (Clot Activator Tubes) and Vacutainer® EDTA tubes for molecular analysis.

Small blood droplets (enough to provide three to four duplicate blood smears) were placed onto clean, pre-labelled microscope slides to make thin blood smears. Blood smears were air-dried and subsequently fixed with absolute methanol for one minute. Once dry, blood smears were stored in slide boxes for further processing in the lab. A modified Giemsa (Fluka, Sigma-Aldrich, Steinheim, Germany) stain solution was prepared with distilled water (ratio of 9:1) in a 50 ml staining container. Air-dried blood smears were stained in the Giemsa solution for 20 min, rinsed with a slow stream of distilled water and again left to air dry.

### Screening of blood smears

Stained smears were examined under the 100× oil immersion objective of a Nikon Eclipse E800 compound microscope (Nikon, Amsterdam, The Netherlands) and digital images of any infections detected were captured with an attached Nikon DS-Fi1 digital camera and accompanying software. Haemoparasites were identified through comparison of morphometric data to previous studies on species of *Hepatozoon* from carnivores [12, 19]. Parasitaemia was calculated per 100 host cells, with ~ 500 host cells (ten fields of 50 host cells) examined per blood smear. Photomicrographs of blood smears were calibrated according to the guidelines stipulated by the ImageJ Image Processing and Analysis software [40]. Measurements of parasites and leopard blood cells were taken with the ImageJ version 1.47 software program (Wayne Rasband National Industries of Health, USA) (http://imagej.nih.gov/ij). All measurements are in micrometres and are given as the range followed by the mean ± standard deviation (SD) in parentheses.

### Molecular analysis

Blood samples collected directly into Vacutainer® EDTA tubes were thawed and used for molecular protocols. DNA was extracted with the KAPA Blood PCR Kit B (Kapa Biosystems, Cape Town, South Africa) according to the protocol provided by the manufacturer. DNA for the dried blood collected from the erythristic leopard was extracted by using the Kapa Express DNA extraction kit (Kapa Biosystems, Cape Town, South Africa), following the manufacturer’s protocols.

Haemogregarine-specific primers 4558F (5ʹ-GCT AAT ACA TGA GCA AAA TCT CAA-3ʹ) and 2733R (5ʹ-CGG AAT TAA CCA GAC AAA T-3ʹ) [41] were used for the detection of *Hepatozoon* species through PCR (polymerase chain reaction) amplification of the *18S* rRNA gene. Fragments of between 995 and 1002 nucleotide (nt) were amplified using the primer set as mentioned above. PCRs were performed with 1.25 µl (10 µM) of each primer, 12.5 µl Kapa Blood Mix B, 7.5 µl molecular grade nuclease-free water (Thermo Fisher Scientific, Vilnius, Lithuania) and 2.5 µl whole blood to make up a final volume of 25 µl per sample. The PCR was undertaken in a Bio-Rad C1000 TouchTM Thermal Cycler PCR machine (Bio-Rad, Hemel Hempstead, UK), under the following conditions. Initial denaturation step of 5 min at 95 °C, followed by 35 cycles of denaturation for 30 s at 95 °C, annealing for 30 s at 50 °C and extension for 1 min at 72 °C. This was followed by a final extension of 7 min at 72 °C, and products were held at 4 °C. Resulting amplicons were visualised on a 1% agarose gel stained with gel red and using a Bio-Rad Gel-DocTM XR+ imaging system (Bio-Rad, Hemel Hempstead, UK) under ultraviolet light.

All positive, purified PCR products were sent for sequencing to Inqaba Biotechnical Industries (Pty) Ltd. (IBSA) (Pretoria, South Africa), a commercial sequencing company, for sequencing in both directions. Resultant sequences species identity was verified against previously published sequences using the Basic Local Alignment Search Tool (BLAST) [42]. Haemogregarine species identity was determined by establishing the closest BLAST match (97–100% to existing sequences available on the GenBank database). All sequences matching *Hepatozoon* spp. were considered positive and, since they were identical within each new species, only one representative sequence of each was included in further analysis.

The software package Geneious R11 (http://www.geneious.com [43]) was used to assemble and edit resultant sequence fragments. Sequences were aligned using the Clustal W 2.1 alignment tool [44] implemented within Geneious R11. A model test was performed using jModelTest 2·1·7 [45], to determine the most suitable nucleotide substitution model, according to the Bayesian information criterion (BIC). The model with the best BIC score was the General Time Reversible [46] model with estimates of invariable sites and a discrete Gamma distribution (GTR+I+G). *18S* rDNA sequences for species of *Hemolivia* Petit, Landau, Baccam & Lainson, 1990, *Hepatozoon* Miller, 1908, *Karyolysus* Labbé, 1894, *Haemogregarina* Danilewsky, 1885 and *Dactylosoma* Labbé, 1894 (parasitising amphibian, reptilian and mammalian hosts) were downloaded from GenBank and aligned with the sequences generated in this study (Table 2). *Adelina dimidiate* Schneider, 1875, *Adelina grylli* Butaeva, 1996 (GenBank: DQ096835-DQ096836) and *Klossiella equi* Smith & Johnson, 1902 (GenBank: MH211602), from the suborder Adeleiorina Léger, 1911 were selected as outgroup. Although eight sequences were obtained from infected leopards, only a single representative of each species of *Hepatozoon* amplified in the present study was used for phylogenetic analyses. Phylogenetic analyses consisted of two datasets, the first alignment a large dataset (*n* = 297) including all representative *H. felis* sequences from GenBank (Additional file 1: Figure S1), and the second alignment based on the results of the first included 57 representative sequences (Table 2). Bayesian inference (BI) was used to infer phylogenetic relationships. The BI analysis was performed using MrBayes 3.2.2 [47] implemented from within Geneious R11. To assess posterior probability support the Markov Chain Monte Carlo (MCMC) algorithm was run for one million generation for the first larger dataset (297 sequences), and 10 million generations for the second the smaller dataset (57 sequences), sampling every 100 generations and using the default parameters. The first 25% of the trees were discarded as ‘burn-in’ with no ‘burn-in’ samples being retained. Results were visualised in Trace, to assess convergence and the ‛burn-inʼ period. Furthermore, uncorrected p-distances for the sequences used were also calculated in PAUP (Phylogenetic Analysis Using Parsimony) version 4.0a152 (Additional file 2: Table S1).

**Table 2:** List of taxa used in the phylogenetic analyses of this study, with associated GenBank accession numbers, host, host family, host common name, country and references

Haemoparasite	Host species	Class	Family	Common name	Country	GenBank ID	Reference
*Hepatozoon americanum*	*Canis familiaris*	Mammalia	Canidae	Domestic dog	USA	AF176836	[41]
*Adelina dimidiata*	*Scolopendra cingulata*	Scolopendromorpha	Scolopendridae	Megarian banded centipede	Bulgaria	DQ096835	[70]
*Adelina grylli*	*Gryllus bimaculatus*	Insecta	Gryllidae	African field cricket	Bulgaria	DQ096836	[70]
*Dactylosoma ranarum*	*Pelophylax* kl. *esculentus*	Amphibia	Ranidae	Edible frog	Canada	HQ224957	[33]
*Haemogregarina balli*	*Chelydra serpentina*	Reptilia	Chelydridae	Common snapping turtle	Canada	HQ224959	[33]
*Haemogregarina pellegrini*	*Malayemys subtrijuga*	Reptilia	Geoemydidae	Mekong snail-eating turtle	Vietnam	KM887508	[82]
*Haemogregarina* sp.	*Pelusios subniger*	Reptilia	Pelomedusidae	East African black mud turtle	Mozambique	KF257925	[78]
*Haemogregarina stepanowi*	*Mauremys caspica*	Reptilia	Geoemydidae	Striped-neck terrapin	Iran	KF257926	[78]
*Hemolivia mariae*	*Egernia stokesii*	Reptilia	Scincidae	Gidgee skink	Australia	KF992711	[79]
*Hemolivia mauritanica*	*Testudo marginata*	Reptilia	Testudinidae	Marginated tortoise	Greece	KF992699	[79]
*Hemolivia parvula*	*Kinixys zombensis*	Reptilia	Testudinidae	Eastern hinged back tortoise	South Africa	KR069082	[34]
*Hemolivia* sp.	*Rhinoclemmys pulcherrima manni*	Reptilia	Geoemydidae	Central American painted wood turtle	Nicaragua	KF992713	[79]
*Hemolivia stellata*	*Rhinella marina*	Amphibia	Bufonidae	Cane toad	Brazil	KP881349	[83]
*Hepatozoon angeladaviesae*	*Philothamnus semivariegatus*	Reptilia	Colubridae	Spotted bush snake	South Africa	MG519502	[89]
*Hepatozoon apri*	*Sus scrofa leucomystax*	Mammalia	Suidae	Japanese boar	Japan	LC314791	[87]
*Hepatozoon ayorgbor*	*Python regius*	Reptilia	Pythonidae	Ball python	Ghana	EF157822	[72]
*Hepatozoon canis*	*Pseudalopex gymnocercus*	Mammalia	Canidae	Pampas fox	Brazil	AY461376	[50]
*Hepatozoon canis*	*Canis lupus familiaris*	Mammalia	Canidae	Domestic dog	Venezuela	DQ439540	[71]
*Hepatozoon canis*	*Canis lupus familiaris*	Mammalia	Canidae	Domestic dog	Israel	KC138535	[25]
*Hepatozoon canis*	*Canis lupus familiaris*	Mammalia	Canidae	Domestic dog	Israel	MH615006	[91]
*Hepatozoon cecilhoarei*	*Philothamnus natalensis natalensis*	Reptilia	Colubridae	Natal green snake	South Africa	MG519504	[89]
*Hepatozoon domerguei*	*Madagascarophis colubrinus*	Reptilia	Lamprophiidae	Madagascar cat-eyed snake	Madagascar	KM234646	[81]
*Hepatozoon felis*	*Felis catus*	Mammalia	Felidae	Domestic cat	Spain	AY620232	[50]
*Hepatozoon felis*	*Felis catus*	Mammalia	Felidae	Domestic cat	Spain	AY628681	[50]
*Hepatozoon felis*	*Panthera leo persica*	Mammalia	Felidae	Asiatic lion	India	HQ829440	[11]
*Hepatozoon felis*	*Panthera pardus fusca*	Mammalia	Felidae	Indian leopard	India	HQ829444	[11]
*Hepatozoon felis*	*Panthera tigris tigris*	Mammalia	Felidae	Bengal tiger	India	HQ829445	[11]
*Hepatozoon felis*	*Felis catus*	Mammalia	Felidae	Domestic cat	Brazil	JN123435	[76]
*Hepatozoon felis*	*Panthera leo persica*	Mammalia	Felidae	Asiatic lion	Thailand	KY056823	[86]
*Hepatozoon fitzsimonsi*	*Kinixys zombensis*	Reptilia	Testudinidae	Eastern hinged back tortoise	South Africa	KR069084	[34]
*Hepatozoon involucrum*	*Hyperolius marmoratus*	Amphibia	Hyperoliidae	Painted reed frog	South Africa	MG041594	[16]
*Hepatozoon ixoxo*	*Sclerophrys pusilla*	Amphibia	Bufonidae	Merten’s Striped Toad	South Africa	MG041604	[16]
*Hepatozoon martis*	*Martes martes*	Mammalia	Mustelidae	European pine marten	Bosnia and Herzegovina	MG136687	[88]
*Hepatozoon silvestris*	*Felis silvestris silvestris*	Mammalia	Felidae	European wild cat	Bosnia and Herzegovina	KX757032	[20]
*Hepatozoon sipedon*	*Nerodia sipedon sipedon*	Reptilia	Colubridae	Northern water snake	Canada	JN181157	[33]
*Hepatozoon* sp.	*Cerdocyon thous*	Mammalia	Canidae	Crab-eating fox	Brazil	AY461377	[50]
*Hepatozoon* sp.	*Martes martes*	Mammalia	Mustelidae	European pine marten	Spain	EF222257	[69]
*Hepatozoon* sp.	*Sciurus vulgaris*	Mammalia	Sciuridae	Eurasian red squirrel	Spain	EF222259	[69]
*Hepatozoon* sp.	*Scelarcis perspicillata*	Reptilia	Lacertidae	Moroccan rock lizard	Morocco	HQ734791	[74]
*Hepatozoon* sp.	*Podarcis vaucheri*	Reptilia	Lacertidae	Andalusian wall lizard	Morocco	HQ734792	[74]
*Hepatozoon* sp.	*Psammophis schokari*	Reptilia	Lamprophiidae	Schokari sand racer	Algeria	KC696565	[77]
*Hepatozoon* sp.	*Meles meles*	Mammalia	Mustelidae	European badger	Spain	KU198330	[84]
*Hepatozoon* sp.	*Canis mesomelas*	Mammalia	Canidae	Black-backed jackal	South Africa	MG919977	[31]
*Hepatozoon* sp.	*Canis mesomelas*	Mammalia	Canidae	Black-backed jackal	South Africa	MG919980	[31]
*Hepatozoon tenuis*	*Afrixalus fornasini*	Amphibia	Hyperoliidae	Greater leaf-folding frog	South Africa	MG041596	[16]
*Hepatozoon theileri*	*Amietia delalandii*	Amphibia	Pyxicephalidae	Delalande’s river frog	South Africa	MG041605	[16]
*Hepatozoon thori*	*Hyperolius marmoratus*	Amphibia	Hyperoliidae	Marbled reed frog	South Africa	MG041602	[16]
*Hepatozoon ursi*	*Ursus thibetanus japonicus*	Mammalia	Ursidae	Asian black bear	Japan	EU041717	[73]
*Hepatozoon ursi*	*Ursus thibetanus japonicus*	Mammalia	Ursidae	Asian black bear	Japan	EU041718	[73]
*Hepatozoon ursi*	*Melursus ursinus*	Mammalia	Ursidae	Sloth bear	India	HQ829437	[75]
*Karyolysus lacazei*	*Lacerta viridis*	Reptilia	Lacertidae	European green lizard	Slovakia	KJ461943	[69]
*Karyolysus latus*	*Podarcis muralis*	Reptilia	Lacertidae	European wall lizard	Slovakia	KJ461939	[69]
*Karyolysus paradoxa*	*Varanus albigularis*	Reptilia	Varanidae	Rock monitor	South Africa	KX011040	[85]
*Karyolysus* sp.	*Zootoca vivipara*	Reptilia	Lacertidae	Viviparous lizard	Poland	KJ461945	[80]
*Klossiella equi*	*Equus ferus caballus*	Mammalia	Equidae	Horse	Canada	MH211602	[90]

## Results

### Taxonomy

**Phylum Apicomplexa Levine, 1970**

**Class Conoidasida Levine, 1988**

**Order Eucoccidiorida Léger & Dubosq, 1910**

**Suborder Adeleorina Léger, 1911**

**Family Hepatozoidae Wenyon, 1926**

**Genus*Hepatozoon*Miller, 1908**

***Hepatozoon luiperdjie*n. sp.**

***Type-host*:***Panthera pardus pardus* (L.) (Carnivora: Felidae).

***Type-locality*:** Lajuma Research Centre (23°02ʹ17.1ʺS, 29°26ʹ26.5ʺE), Limpopo Province, South Africa.

***Other localities*:** Greater Kruger Conservation Area (24°34ʹ43.50ʺS, 31°25ʹ46.48ʺE), Mpumalanga Province; Lydenburg area (25°9ʹ51.31ʺS, 30°26ʹ55.46ʺE), Mpumalanga Province, South Africa.

***Type-material*:** Hapantotype, 1 peripheral blood smear from the type-host *P. p. pardus* and type-locality (23°02ʹ17.1ʺS, 29°26ʹ26.5ʺE), deposited under the accession number NMBP392 in the protozoan collection of the National Museum, Bloemfontein, South Africa.

***Vector*:** Unknown.

***Representative DNA sequences*:** Two sequences, of a 995 nt fragment of the *18S* rRNA gene of *Hepatozoon luiperdjie* n. sp., isolated from the type-host *P. p. pardus,* deposited under the accession numbers MN793002 and MN793003 in the GenBank database.

***ZooBank registration*:** To comply with the regulations set out in article 8.5 of the amended 2012 version of the *International Code of Zoological Nomenclature* (ICZN) [48], details of the new species have been submitted to ZooBank. The Life Science Identifier (LSID) of the article is urn:lsid:zoobank.org:pub:9E65A924-729F-43A5-AE1F-8001204B6A6A. The LSID for the new name *Hepatozoon luiperdjie* n. sp. is urn:lsid:zoobank.org:act:2293B0B3-3B91-4BDC-8246-50215B80F8D5.

***Etymology***: The species epithet is derived from the Afrikaans language diminutive name for the host *P. p. pardus*, which in Afrikaans is referred to as “luiperdjie”.

**Description**

**Fig. 1:**
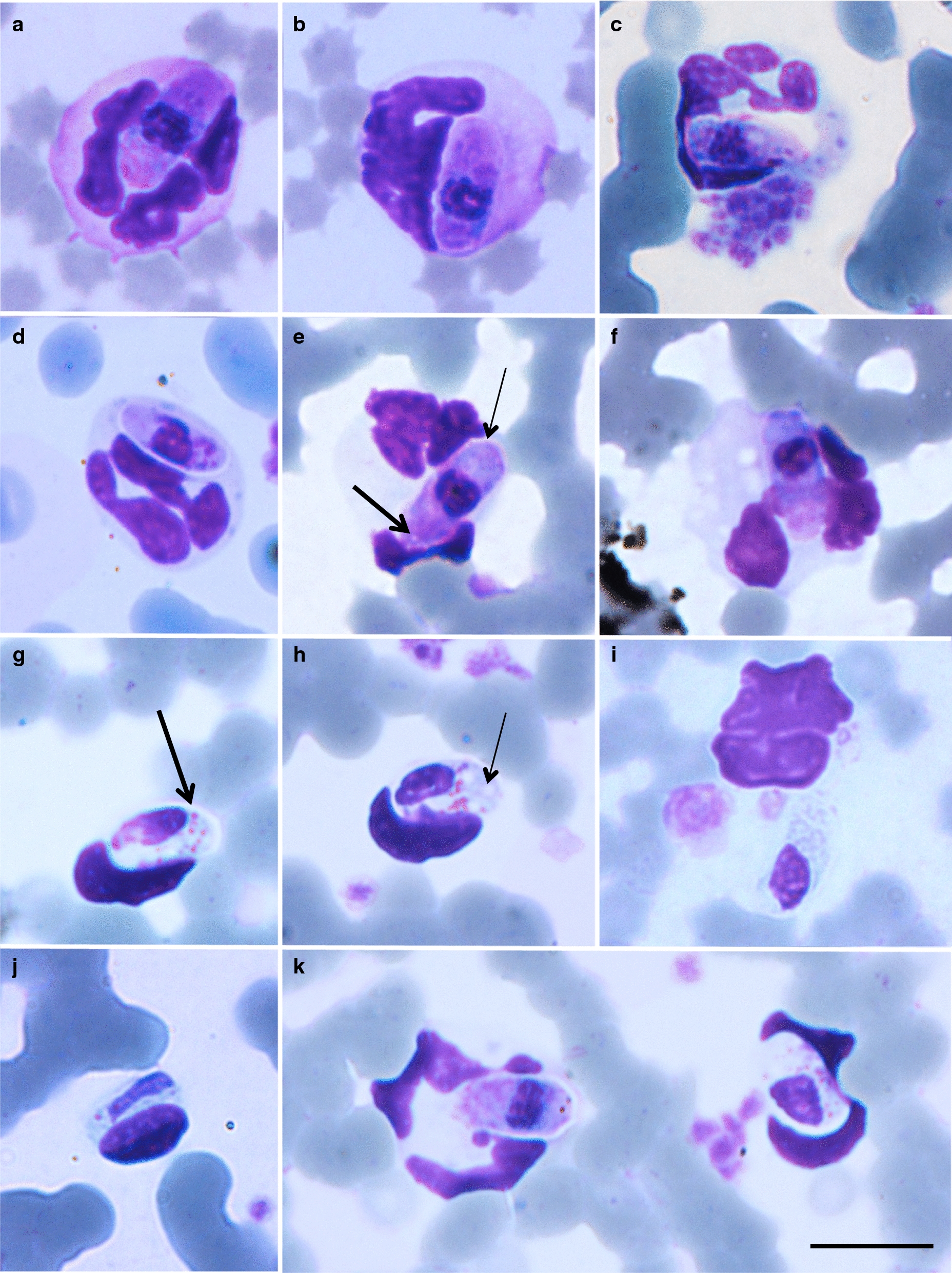
**a–f** Peripheral blood gamont stages of *Hepatozoon luiperdjie* n. sp. in the African leopard *Panthera pardus pardus* from hapantotype slide (NMBP392). **a**, **b**, **e**, **f** Mature gamonts within neutrophils, where enlargement of host cell and displacement of host cell nucleus is apparent. **c** Extracellular gamont. **d** Immature gamont. **e** Mature gamont in which small posterior vacuoles (thick arrow) and thin capsule (thin arrow) can be seen. **c**, **f** Disintegration of neutrophils by infecting gamonts. **g**–**k** Peripheral blood gamont stages of *Hepatozoon ingwe* n. sp. in the African leopard *Panthera pardus pardus* from hapantotype slide (NMBP393). **g**, **h**, **k** Mature gamonts within lymphocytes, where lateral compression of host cell is apparent. **g** Mature gamont in which bright pink granules and thin capsule (thick arrow) can be seen. **h** Mature gamont with prominent posterior vacuoles (thin arrow). **i** Extracellular gamont. **j** Immature gamont. **k** Co-infection of *Hepatozoon luiperdjie* n. sp. (on the left) and *Hepatozoon ingwe* n. sp. in the same leopard. *Scale-bar*: 10 µm

***Gamonts.*** Most abundant stage in peripheral blood smears (Fig. 1a-f). Extracellular forms (Fig. 1c) and immature gamonts (Fig. 1d) rarely observed and no division stages detected. Mature gamonts measure 9.9–12.6 × 4.1–5.0 (11.0 ± 0.9 × 4.7 ± 0.4) (*n* = 53), area of 39.5–46.2 (42.0 ± 2.9) µm^2^ (*n* = 53). Mature gamonts mostly conspicuous within neutrophil cytoplasm (Fig. 1a–c), sometimes hardly visible and concealed by host cell nucleus; elongate, with bluntly rounded extremities, thin visible capsule (Fig. 1a); cytoplasm stained pale purple with some gamonts containing bright magenta and basophilic staining granules (Fig. 1a, f). Some gamonts with 2 to 3 small, slightly noticeable posteriorly situated vacuoles (Fig. 1e, thick arrow). Gamont nuclei measure 3.0–3.6 × 3.1–3.8 (3.5 ± 0.3 × 3.4 ± 0.3) (*n* = 53), area of 8.8–9.6 (9.2 ± 0.3) µm^2^ (*n* = 53); rounded and acentric, usually as wide as gamont at widest point, mostly located closer to anterior than posterior of gamont and stained dark purple, with densely stranded chromatin. Capsules 0.3–0.7 (0.5 ± 0.2) thick (*n* = 53) and observable in most gamonts (Fig. 1e, thin arrow).

***Prevalence and parasitaemia.****Hepatozoon luiperdjie* n. sp. occurred in peripheral blood of 8/16 (prevalence 50%) individual *P. p. pardus* sampled. This haemogregarine formed co-infections with *Hepatozoon ingwe* n. sp. (see below) in 6 out of 16 leopards (prevalence of 38%), and was the sole species of *Hepatozoon* detected in 2 out of 16 individuals (prevalence of 13%). Prevalence of 63% (5/8) in males and 38% (3/8) in females infected. No captive individuals infected by this haemogregarine. Parasitaemia varied between individuals and could only be determined in WM1 (11.3%), WF1 (21.5%), WM3 (1.9%), WF3 (15.4%) and WM4 (4.8%). Average parasitaemia in all five leopards was 11.0%.

***Effect on host cells.*** Gamonts sometimes compressed the lobulated nucleus of neutrophils, either towards the periphery of the host cell (Fig. 1a), or towards one side (Fig. 1b). Parasitized neutrophils measured 12.7–14.3 × 12.1–14.1 (13.6 ± 0.8 × 13.1 ± 0.7) (*n* = 53), area of 130.2–186.0 (146.3 ± 22.9) µm^2^ (*n* = 53). Healthy, uninfected neutrophils measured 7.6–14.4 × 7.0–13.0 (11.1 ± 1.5 × 10.3 ± 1.3) (*n* = 450), area of 46.3–127.2 (89.1 ± 18.3) µm^2^ (*n* = 450). Nuclei of parasitized neutrophils measured 12.8–25.3 × 3.3–13.3 (19.8 ± 5.2 × 5.7 ± 4.2) (*n* = 53), area of 53.3–116.9 (68.8 ± 27.0) µm^2^ (*n* = 53). Dimensions of healthy neutrophil nuclei were 10.3–29.3 × 1.7–4.5 (20.5 ± 3.9 × 2.7 ± 0.6) (*n* = 450), area of 25.1–58.1 (41.7 ± 7.4) µm^2^ (*n* = 450). Thus, infected neutrophils were slightly longer and wider, with larger surface area. Nuclei of infected neutrophils were slightly longer and narrower, with greater surface area.

**Remarks**

Prior to this study, *Hepatozoon* spp. reported from wild carnivores were only *H. felis*, *H. canis* and mostly as an unidentified species of *Hepatozoon* [6, 10–12, 22, 24, 25, 29, 49–63], except by Hodžić et al. [20] who recently described *Hepatozoon silvestris* Hodžić, Alić, Prašović, Otranto, Baneth & Duscher, 2017 in an European wildcat from eastern Europe. Infection with a morphologically and genetically distinct *Hepatozoon* sp. was confirmed by our study in wild African leopards in various areas throughout South Africa, both in male and female hosts. The haemogregarine described here appears to develop only gamont stages in the peripheral blood of *P. p. pardus*. Therefore, with no division stages detected, it was placed within the genus *Hepatozoon.*

*Hepatozoon luiperdjie* n. sp. was on average longer than *H. canis* from *Cerdocyon thous* (L.) in Brazil [51], and longer, but with a similar average width to *H. felis* from *Felis catus* (L.) in Israel [19], therefore within the relative morphometrical range of both species (Table 3). This haemogregarine was morphometrically most similar to an unnamed species of *Hepatozoon* detected in *Lynx rufus* (Schreber) from the USA [57]. It also differed from the other species of *Hepatozoon* detected in the monocytes of leopards during our study. The most striking feature of *H. luiperdjie* n. sp. is the densely chromatisized, acentric nucleus, relatively smaller than that of *H. felis* [19, 20]. *Hepatozoon luiperdjie* n. sp. also seemed to exclusively infect the neutrophils of the host. The life-cycle of this parasite remains to be determined.

**Table 3:** Details and measurements for *Hepatozoon luiperdjie* n. sp. and *Hepatozoon ingwe* n. sp. and closely related *Hepatozoon* species in wild and domestic carnivores

*Hepatozoon* spp.	Host species	Country	Host cells infected	GenBank ID	Gamont dimensions L × W in µm^a^ (LW in µm^2^)	Gamont nuclei dimensions L × W in µm^a^ (LW in µm^2^)	*n*	Reference
*H. canis*	*Canis familiaris*	India	Neutrophils	–	9.5–11.8 × 5.1–6.0	–	–	[92]
*H. canis*	*Cerdocyon thous*	Brazil	Leukocytes	–	11.4 × 5.4 (45.9)	–	–	[51]
*H. canis*	*Cerdocyon thous*	Brazil	Neutrophils	–	9.1 ± 0.5 × 5.3 ± 0.5	–	–	[61]
*H. felis*	*Felis catus*	Israel	Neutrophils.	KC138534	10.5 ± 0.6 × 4.7 ± 0.8	4.0 ± 0.3 × 3.2 ± 0.5	13	[19]
*H. felis*	*Felis silvestris*	Bosnia and Herzegovina	Extracellular	KX757033	10.5 ± 0.4 × 4.4 ± 0.4	4.7 ± 0.3 × 4.4 ± 0.3	–	[20]
*H. ingwe* n. sp.	*Panthera pardus pardus*	South Africa	Lymphocytes	MN793000; MN793001	11.4 ± 1.2 × 4.8 ± 0.2 (44.2 ± 4.4)	5.1 ± 0.6 × 3.0 ± 0.6 (12.2 ± 3.3)	87	This study
*H. luiperdjie* n. sp.	*Panthera pardus pardus*	South Africa	Neutrophils	MN793002; MN793003	11.0 ± 0.9 × 4.7 ± 0.4 (42.0 ± 2.9)	3.5 ± 0.3 × 3.4 ± 0.3 (9.2 ± 0.3)	53	This study
*H. silvestris*	*Felis silvestris*	Bosnia and Herzegovina	Extracellular	KX757032	11.7 ± 0.5 × 5.2 ± 0.7	6.3 ± 1.3 × 3.0 ± 0.8	11	[20]
*Hepatozoon* sp.	*Felis catus*	Brazil	Neutrophils	–	9.9 ± 0.4 × 5.3 ± 0.2 (45.9 ± 4.9)	–	–	[65]
*Hepatozoon* sp.	*Prionailurus bengalensis*	Thailand	Neutrophils	GQ926902	9.8 ± 0.4 × 5.2 ± 0.4	–	10	[54]
*Hepatozoon* sp.	*Leopardus pardalis*	Brazil	Neutrophils	EU028344	7.4 × 4.2 (27.0)	–	1	[35]
*Hepatozoon* sp.	*Lynx rufus*	USA	Leukocytes	–	11.0 × 2.5	–	–	[57]
*Hepatozoon* sp.	*Panthera pardus ciscaucasica*	Iran	Neutrophils	–	11.4 ± 0.3 × 5.2 ± 0.2 (39.5 ± 3.2)	–	–	[12]

^a^Mean ± standard deviation (SD)

*Abbreviations*: n, number measured; L, length; W, width

***Hepatozoon ingwe*n. sp.**

***Type-host*:***Panthera pardus pardus* (L.) (Carnivora: Felidae).

***Type-locality*:** Lajuma Research Centre (23°02ʹ17.1ʺS, 29°26ʹ26.5ʺE), Limpopo Province, South Africa.

***Other localities*:** Greater Kruger Conservation Area (24°34ʹ43.50ʺS, 31°25ʹ46.48ʺE), Mpumalanga Province; captive facility (24°30ʹ52.44ʺS, 30°54ʹ8.82ʺE), Mpumalanga Province; Lydenburg area (25°9ʹ51.31ʺS, 30°26ʹ55.46ʺE), Mpumalanga Province, South Africa.

***Type-material*:** Hapantotype, 1 peripheral blood smear from the type-host *P. p. pardus* and type-locality (23°02ʹ17.1ʺS, 29°26ʹ26.5ʺE), deposited under accession number NMBP393 in the protozoan collection of the National Museum, Bloemfontein, South Africa.

***Vector*:** Unknown.

***Representative DNA sequences*:** Two sequences, representing a 995 nt fragment of the *18S* rRNA gene of *Hepatozoon ingwe* n. sp., isolated from the type-host *P. p. pardus,* deposited under the accession numbers MN793000 and MN793001 in the GenBank database.

***ZooBank registration*:** To comply with the regulations set out in article 8.5 of the amended 2012 version of the *International Code of Zoological Nomenclature* (ICZN) [48], details of the new species have been submitted to ZooBank. The Life Science Identifier (LSID) of the article is urn:lsid:zoobank.org:pub:9E65A924-729F-43A5-AE1F-8001204B6A6A.The LSID for the new name *Hepatozoon ingwe* n. sp. is urn:lsid:zoobank.org:act:65A2DC3D-BABB-443A-82A7-DBCFFCAE3636.

***Etymology*:** The species epithet is derived from that of the Zulu language name for the host *P. p. pardus*, which in Zulu is referred to as “ingwe”. Noun in apposition.

**Description**

***Gamonts***. Most abundant stage in peripheral blood smears (Fig. 1g–k). Extracellular forms (Fig. 1i) and immature gamonts (Fig. 1j) rarely observed, no division stages detected. Mature gamonts measure 9.8–12.6 × 4.5–5.0 (11.4 ± 1.2 × 4.8 ± 0.2) (*n* = 87), surface area of 38.7–48.9 (44.2 ± 4.4) µm^2^ (*n* = 87); mostly visible within leukocyte cytoplasm (Fig. 1g, h, k), but in some cases gamonts were concealed by leukocyte nucleus; elongate with round extremities, cytoplasm stained pale blue, slight granulation, minimal basophilic stippling anteriorly; cytoplasm contained bright pink staining granules (Fig. 1g, h). Gamonts with thin visible capsules (Fig. 1g, thick arrow) and 2 to 4 prominent vacuoles posteriorly situated (Fig. 1h, thin arrow); gamont nuclei measure 4.4–5.7 × 2.5–3.7 (5.1 ± 0.6 × 3.0 ± 0.6) (*n* = 87), area of 8.7–16.5 (12.2 ± 3.3) µm^2^ (*n* = 87). Nuclei stained dark purple with loosely stranded chromatin, through which parts of cytoplasm were often visible (Fig. 1g, h). Nuclei elongate, usually narrower than gamont at widest point, mostly anteriorly located. Capsule measured 0.4–0.7 (0.5 ± 0.1) thick (*n* = 87) and observable in most gamonts.

***Prevalence and parasitaemia.*** Detected in the peripheral blood of 7 out of 16 individual *P. p. pardus* sampled (prevalence of 44%). Of these 6 out of 16 leopards had co-infections with *H. luiperdjie* n. sp. (prevalence of 38%) and 1 out of 16 individuals was solely infected with this species of *Hepatozoon* (prevalence 6%). More males than females were infected, prevalence 50% (4/8) of males and 38% (3/8) of females. One captive female was infected (prevalence in captivity 13% or 1/8). Parasitaemia varied between individuals and could only be determined in WM1 (32%), WF1 (57%), WF3 (13%), WM4 (21%) and WM5 (7%). Average parasitaemia was 30.8%. Gamonts not observed in smears of CF5; however, prevalence confirmed by PCR amplification.

***Effect on host cells.*** Gamonts usually compacted lymphocyte nuclei towards one side and completely usurped lymphocyte cytoplasm (Fig. 1g, h). Parasitized lymphocytes measured 10.9–11.6 × 8.4–9.7 (11.3 ± 0.3 × 9.1 ± 0.6) (*n* = 87), area of 73.6–83.4 (79.2 ± 4.3) µm^2^ (*n* = 87). Healthy, uninfected lymphocytes measured 6.8–15.7 × 6.1– 13.9 (10.9 ± 2.1 × 9.7 ± 1.9) (*n* = 261), area of 36.6–156.8 (81.7 ± 29.4) µm^2^ (*n* = 261). Nuclei of parasitized lymphocytes measured 8.4–11.9 × 3.8–5.7 (10.0 ± 1.5 × 4.78 ± 0.8) (*n* = 87), area of 30.2–36.9 (33.5 ± 3.0) µm^2^ (*n* = 87). Dimensions of healthy lymphocyte nuclei were 6.1–16.1 × 4.2–10.7 (9.7 ± 2.2 × 7.3 ± 1.4) (*n* = 261), area of 26.2–92.1 (56.2 ± 15.4) µm^2^ (*n* = 261). Thus, infected lymphocytes were slightly longer and narrower, with smaller surface area. Infected lymphocyte nuclei measured slightly longer and narrower, with smaller surface area.

**Remarks**

This haemogregarine appears to develop only gamont stages in the peripheral blood of *P. p. pardus*, and with no division stages detected it was placed within *Hepatozoon. Hepatozoon ingwe* n. sp. measured within the same range size as *H. luiperdjie* n. sp. described above. However, *H. ingwe* n. sp. seemed to exclusively infect the lymphocytes of the host, unlike the gamonts of *H. luiperdjie* n. sp. *Hepatozoon ingwe* n. sp. was morphometrically similar to an unnamed *Hepatozoon* detected in the neutrophils of *P. p. ciscaucasica* from Iran [12] (Table 3). This haemogregarine also measured longer and wider than *H. felis* from domestic cats in Israel [19], widely considered a redescription of *H. felis* (Table 3). *Hepatozoon ingwe* n. sp. was significantly longer (*P* = 0.0064) and somewhat wider (*P* = 0.3023), with a comparatively larger surface area than that of *H. luiperdjie* n. sp. (*P* = 0.0593) (Table 3). Characteristic features of *H. ingwe* n. sp. include the pale blue staining cytoplasm containing bright pink staining granules, prominent vacuoles at the posterior, and elongated nuclei similar to that of *H. canis*. The life-cycle of this parasite remains to be determined.

### Differential diagnoses

Each species of haemogregarine described here infected a particular type of leukocyte: *H. luiperdjie* n. sp. were exclusively found in neutrophils and *H. inwe* n. sp. in lymphocytes. The parasitaemia of *H. ingwe* n. sp. (30.8%), and *H. luiperdjie* n. sp. (11.0%), was higher than the range of 0.1–4.0% reported by other studies on domestic cats in Israel [64], an ocelot *Leopardus pardalis* (L.) in Brazil [35], a Tsushima leopard cat *Prionailurus bengalensis* (Kerr) in Thailand [54], Iriomote cats *P. b. iriomotensis* (Imaizumi) in Japan [52] and a Tsushima leopard cat *P. bengalensis* in Japan. The parasitaemia of *H. ingwe* n. sp. was also higher than that of *H. luiperdjie* n. sp., possibly indicating that leopard host immune system may be better at suppressing infections by *H. luiperdjie* n. sp. in neutrophils, than *H. ingwe* n. sp. in lymphocytes. Additionally, there seemed to be a clear association between sex and parasitaemia in this study, with average parasitaemia in females (*H. luiperdjie* n. sp. parasitized 18.2% and *H. ingwe* n. sp. parasitized 35% of host cells) higher than that in males (*H. luiperdjie* n. sp. parasitized 5.99% and *H. ingwe* n. sp. parasitized 20% of host cells). This may be a noticeable health concern, since it is still unclear whether feline hepatozoonosis can be transferred within the uterus. However, this trend in parasitaemia needs further investigation over a larger variety and number of felid hosts.

In addition to infecting different types of host cells, the two new species showed clear morphological distinctions of peripheral blood gamont stages on a morphometric basis and differences in staining properties. The gamonts of *H. luiperdjie* n. sp. are shorter, thinner and with a smaller surface area than those of *H. ingwe* n. sp. Furthermore, the staining properties of their cytoplasm, the marked presence of vacuoles in *H. ingwe* n. sp. and the morphometric and staining differences in their nuclei. In terms of nucleus dimensions, the nucleus of *H. luiperdjie* n. sp. was significantly shorter (*P* < 0.0001*)*, broader (*P* < 0.0001) and larger than that of *H. ingwe* n. sp. (*P* = 0.0025). Both species described here had a larger surface area compared to the unnamed species of *Hepatozoon* parasitizing neutrophils of *Leopardus pardalis* from Brazil [35] and an unnamed species of *Hepatozoon* parasitising neutrophils of *P. p. ciscaucasica* from Iran [12] (Table 3). While both new species of *Hepatozoon* had a smaller surface area as compared to an unnamed species of *Hepatozoon* from domestic cats [65] and *H. canis* from *Cerdocyon thous* from Brazil, respectively [61] (Table 3). Data on the morphometrics of large numbers of gamonts are scarce, with some studies measuring only a few gamonts [19, 20], or even only a single gamont [35]. Our study focused on screening live hosts, while most other studies analysed necropsied or biopsied samples [19, 20, 66], or did not focus on morphological descriptions of the gamont stage [10, 11, 22, 24, 27, 29, 31, 52, 53, 55, 58, 64, 67–69]. Several of these studies reported only on molecular detection due to the general low parasitaemia of these haemogregarines [66], as confirmed by the absence of gamonts in the peripheral blood smear of CF5 in our study.

### Molecular analyses

**Fig. 2:**
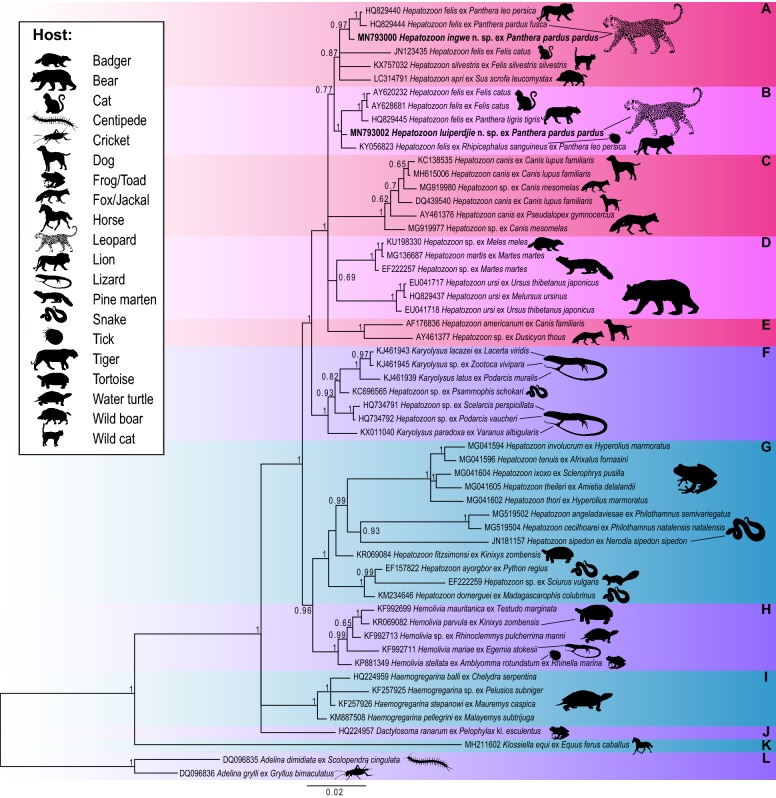
Bayesian inference (BI) phylogram based on *18S* rDNA sequences. Phylogram illustrating the phylogenetic relationships between *Hepatozoon luiperdjie* n. sp. and *Hepatozoon ingwe* n. sp. (shown in bold) with 55 representative sequences of other species of *Dactylosoma*, *Haemogregarina*, *Hepatozoon*, *Karyolysus* and *Hemolivia* retrieved from GenBank. *Adelina dimidiata*, *Adelina grylli* and *Klossiella equi* were selected as the outgroup. Posterior probability values lower than 0.60 were omitted. The scale-bar represents 0.02 nucleotide substitutions per site. Distinct clades are presented in alternating colors and letters A to L highlight 12 distinct clades

Amplicons were derived from *H. luiperdjie* n. sp. and *H. ingwe* n. sp. from the blood of leopards and the details of all sequences used for analysis are presented in Table 2. Although eight sequences were obtained from infected leopards, only a single sequence per species of *Hepatozoon* was used in the phylogenetic analysis (Fig. 2), as sequences obtained from samples with single species infections were identical for the respective new species. Sequences obtained from leopards with mixed *Hepatozoon* infections were not included in the phylogenetic analysis, as sequences contained a double chromatogram peak or two separate bases called at the same position (heterozygous positions) from the two new species of *Hepatozoon* amplified. Based on the uncorrected p-distance for the *18S* rRNA gene between *H. ingwe* n. sp. and *H. felis* [amplified from the Asiatic lion, *Panthera leo persica* (L.) (GenBank: HQ829440) and Indian leopard, *Panthera pardus fusca* (L.) (GenBank: HQ829444)] interspecific divergence was 1.0% (Additional file 2: Table S1). *Hepatozoon luiperdjie* n. sp. and *H. felis* amplified from domestic cats, *F. catus* (GenBank: AY620232 and AY628681) and from a Bengal tiger, *P. tigris tigris* (L.) (GenBank: HQ829445) had an interspecific divergence of 1.0% (Additional file 2: Table S1). The interspecific divergence between *H. luiperdjie* n. sp. and *H. ingwe* n. sp. was 1.0%.

For the BI phylogenetic analyses, first a large dataset was used comprising 297 sequences (Additional file 1: Figure S1). This analysis included all the available *18S* rDNA sequence data of *H. felis* downloaded from GenBank. The genera *Hemolivia*, *Hepatozoon* (parasitising amphibian, reptilian and rodent hosts), *Karyolysus*, *Haemogregarina* and *Dactylosoma* formed separate and well-supported clades at the base of the phylogeny (Additional file 1: Figure S1). Species of *Karyolysus* and several species most likely incorrectly identified as species of *Hepatozoon*, formed a sister group to a large clade comprising species of *Hepatozoon* from large mammals. The phylogenetic analysis showed *H. felis* as paraphyletic forming several clusters, along with *Hepatozoon americanum* Vincent-Johnson, MacIntire, Lindsay, Lenz, Baneth, Shkap & Blagburn, 1997, *Hepatozoon apri* Yamamoto, Tokiwa, Tobiume, Akamatsu, Matsuo, Moribe & Ike, 2017, *Hepatozoon canis*, *Hepatozoon martis* Hodžić, Alić, Beck, Beck, Huber, Otranto, Baneth & Duscher, 2018, *Hepatozoon silvestris*, *Hepatozoon ursi* Kubo, Uni, Agatsuma, Nagataki, Panciera, Tsubota, Nakamura, Sakai, Masegi & Yanai, 2008 and *H. luiperdjie* n. sp. and *H. ingwe* n. sp. Although several sequences are identified or designated as *H. felis*, we consider the sequences isolated from *H. felis* in domestic cats from Spain (GenBank: AY620232; AY628681) as sufficient representatives of *H. felis* based on phylogenetic comparisons to sequences in the formal redescription and molecular characterisation of *H. felis* [19].

The second BI phylogenetic analysis was based on 57 *18S* rDNA sequences (Fig. 2). Species of *Hepatozoon* isolated from large mammal hosts formed a large well-supported clade (Clades A-E, Fig. 2). Clades A and B (monophyletic group), and Clades C, D and E, formed a polytomy of four distinct groups. In Clade A, *H. ingwe* n. sp. was shown as a sister taxon to a well-supported monophyletic cluster of *H. felis* in big cats from India, namely the Asiatic lion (GenBank: HQ829440) and Indian leopard (GenBank: HQ829444) (Clade A in Fig. 2). Furthermore, *H. silvestris* (GenBank: KX757032), isolated form the European wild cat *Felis silvestris silvestris* Schreber, and *H. apri* (GenBank: LC314791) isolated from the Japenese boar, *Sus scrofa leucomystax* Temminck formed a polytomy at the base of clade A. In clade B, *H. luiperdjie* n. sp. clustered with sequences isolated from *H. felis* in domestic cats from Spain (GenBank: AY620232; AY628681), a bengal tiger, *P. tigris tigris* (GenBank: HQ829445) from India, and *H. felis* amplified from a tick *Rhipicephalus sanguineus* (Latreille) that was collected from an Asiatic lion *Panthera leo persica* (GenBank: KY056823). Clade C was a monophyltic clade of *H. canis* and species of *Hepatozoon* isolated from various canid hosts. In clade D, *H. martis* isolated from the European badger *Meles meles* (L.) and the European pine marten *Martes martes* (L.), formed a sister group to *H. ursi* isolated from the Japanese black bear *Ursus thibetanus japonicus* Schlegel and the sloth bear *Melursus ursinus* (Shaw). Clade E comprised *H. americanum*, isolated from domestic dogs, *Canis familiaris* L. and an unnamed species of *Hepatozoon* in crab-eating foxes *Dusicyon thous azarae* (L.).

## Discussion

Since the late 1960’s, the African leopard has been a favoured research subject of ethologists and ecologists. However, research on haematozoans of large carnivores is sparse and often occurs as non-specific reports, and according to Peirce et al. [6] researchers often automatically identify species of *Hepatozoon* in African carnivores as *H. canis* or *H. felis*. According to Baneth et al. [19], most studies on hepatozoonosis have emphasized the detection of the parasite, with little attention given to other aspects such as transmission and epidemiology. It is therefore not unexpected that the scant research on health aspects of African leopards left gaps in knowledge of especially their haemoparasites. Our study addressed these gaps and confirmed co-infection of two morphologically and genetically distinct *Hepatozoon* species in wild and captive African leopards in various areas throughout South Africa, both in male and female hosts.

No leopards sampled during our study displayed any clinical symptoms associated with hepatozoonosis, confirming similar reports by authors such as Brocklesby & Vidler [62], Averbeck et al. [22] and East et al. [25]. Prevalence of *Hepatozoon* varies between hosts and regions. The overall prevalence of hepatozoonosis recorded in this study was 56%, which is similar to that reported in Iriomote cats *Prionailurus bengalensis iriomotensis* from Japan (56.7%) [55], spotted hyenas *Crocuta crocuta* (Erxleben) from Zambia (56%) [28], and captive Asiatic lions *P. l. persica* from India (55.56%) [11]. Prevalence found during our study was slightly higher than the prevalence of unknown species of *Hepatozoon* reported from Indian leopards *P. p. fusca* (50%) [11] and much higher than that of African wild dogs *Lycaon pictus* (Temminck) in South Africa (0.7%) [31]. The prevalence of *H. luiperdjie* n. sp. (50%) was similar to that of an unknown species of *Hepatozoon* reported from Indian leopards *P. p. fusca* in India (50%) [11], and it was lower than the prevalence of *H. ingwe* n. sp. (44%). The prevalence of both new species described in this study was higher in males than females, with 63% of males infected with *H. luiperdjie* n. sp. and 50% of males infected with *H. ingwe* n. sp., and 38% of females infected with *H. luiperdjie* n. sp. and *H. ingwe* n. sp., respectively.

The two new haemogregarines had dissimilar effects on their respective host cells. *Hepatozoon luiperdjie* n. sp. caused enlargement of neutrophil cells and their nuclei and *H. ingwe* n. sp. reduced the size of lymphocytes and condensed their nuclei. Although Baneth et al. [19] reported on a species of *Hepatozoon* that infects both neutrophils and lymphocytes, our study showed that co-infecting species of *Hepatozoon* can inhabit different types of leukocytes, with different effects and morphological characteristics of their gamont stages.

It is evident based on morphological and molecular data that *H. luiperdjie* n. sp. and *H. ingwe* n. sp. are distinct species. These species are also distantly related to *H. felis* (GenBank: AY628681) based on *18S* rDNA sequence comparisons, isolated by Criado-Fornelio et al. [50] from domestic cats from Spain. The *H. felis* isolates from Spain [50] are widely regarded as the representative *H. felis* isolates to be used for comparison [25, 55, 60]. Thus, based on the phylogenetic relationships and comparisons of *H. felis* and *H. felis-*like species of *Hepatozoon,* the identity of the *Hepatozoon* infecting large carnivores, currently identified as *H. felis* by Pawar et al. [11] is questioned. Therefore, based on these genetic distinctions, as well as morphological characteristics and effect on host cells, the two haemogregarines described here were deemed to be two different species and new to science. The phylogenetic results from our study showed *H. luiperdjie* n. sp. and *H. ingwe* n. sp. as distinct species as compared to the currently recognised species of *Hepatozoon* infecting large mammal hosts, i.e. *H. americanum*, *H. apri*, *H. canis*, *H. felis*, *H. martis*, *H. silvestris* and *H. ursi.* We therefore suggest that these haemogregarines may need to be re-classified based on morphological, morphometric and molecular analysis.

Prior to our study, only *H. felis*, *H. canis* and several unknown species of *Hepatozoon* have been reported from African carnivores [6, 8, 10, 22, 24, 28, 31, 57, 64, 65], but this study confirmed a mixed population of two genetically distinct haemogregarines from both captive and wild leopards, across males and females and from leopards representative from three core populations as identified by Daly et al. [36]. The topology of our BI tree confirmed the suggestion of Hodžić et al. [20], that *H. felis* should be viewed as a species complex.

## Conclusions

As shown in this study, morphology and the effect on host cells are important parameters that should be taken into account when identifying species of *Hepatozoon*. By using different techniques of identification, a better understanding of the parasite and its relation to its host may become possible. Thus, *Hepatozoon* species identified as either *H. felis* or *H. canis* based on the host parasitized should be re-evaluated using both morphological and molecular characteristics, as well as the type of and effect on the host cells. The value of the results from our study, in addition to describing two new haemogregarine species, is that we present results obtained from live animals in the wild. It is important to identify these parasites to species level in order to better understand potential zoonotic effects on different host species, and to further investigate the possible transfer of haemogregarines from wild to domestic animals. In addition, this paper provides valuable criteria to be considered when describing *Hepatozoon* infections from wild carnivores. Possible future work on these haemogregarines should include in depth investigations on the life cycles and vectors of these species.

## Supplementary information

**Additional file 1: Figure S1.** Bayesian inference (BI) phylogram based on 297 *18S* rDNA sequences illustrating the phylogenetic relationships between *H. felis*, *H. luiperdjie* n. sp. and *H. ingwe* n. sp. (shown in bold) and other species of *Dactylosoma*, *Haemogregarina*, *Hepatozoon*, *Karyolysus* and *Hemolivia* retrieved from GenBank. *Adelina dimidiate*, *A. grylli* and *Klossiella equi* were selected as the outgroup. The scale-bar represents 0.02 nucleotide substitutions per site.

**Additional file 2: Table S1.** Estimates of divergence using partial *18S* rDNA sequences from the haemogregarines species used in the current study. Distance matrix showing ranges as percentage for the genetic p-distances between the sequences. Alignment length 1924 nt.

## Data Availability

Data supporting the conclusions of this article are included within the article and its additional files. The datasets used and analysed during this study will be made available by the corresponding author upon reasonable request. The newly generated sequences were submitted to the GenBank database under the accession numbers MN792996-MN792999 (*Hepatozoon* spp. mixed infections), MN792000-MN792001 (*H. ingwe* n. sp.), and MN792002-MN792004 (*H. luiperdjie* n. sp.).

## Abbreviations

BLAST: Basic Local Alignment Search Tool
BI: Bayesian inference
BIC: Bayesian information criterion
GTR+I+G: general time reversible model with estimates of invariable sites and a discrete Gamma distribution
ICZN: International Code of Zoological Nomenclature
IUCN: International Union for the Conservation of Nature
LSID: Life Science Identifier
MCMC: Markov Chain Monte Carlo
NCBI: National Center for Biotechnology Information
PAUP: Phylogenetic Analysis Using Parsimony
PCR: polymerase chain reaction
NRF: South African National Research Foundation
UESM: Unit for Environmental Sciences and Management
