# Supplementary figures and images for: Molecular characterisation and morphological description of two new species of Hepatozoon Miller, 1908 (Apicomplexa: Adeleorina: Hepatozoidae) infecting leukocytes of African leopards Panthera pardus pardus (L.)
Source: Parasit Vectors. 2020 May 1;13:222. doi: 10.1186/s13071-020-3933-6 (PMC7195708; doi:10.1186/s13071-020-3933-6)

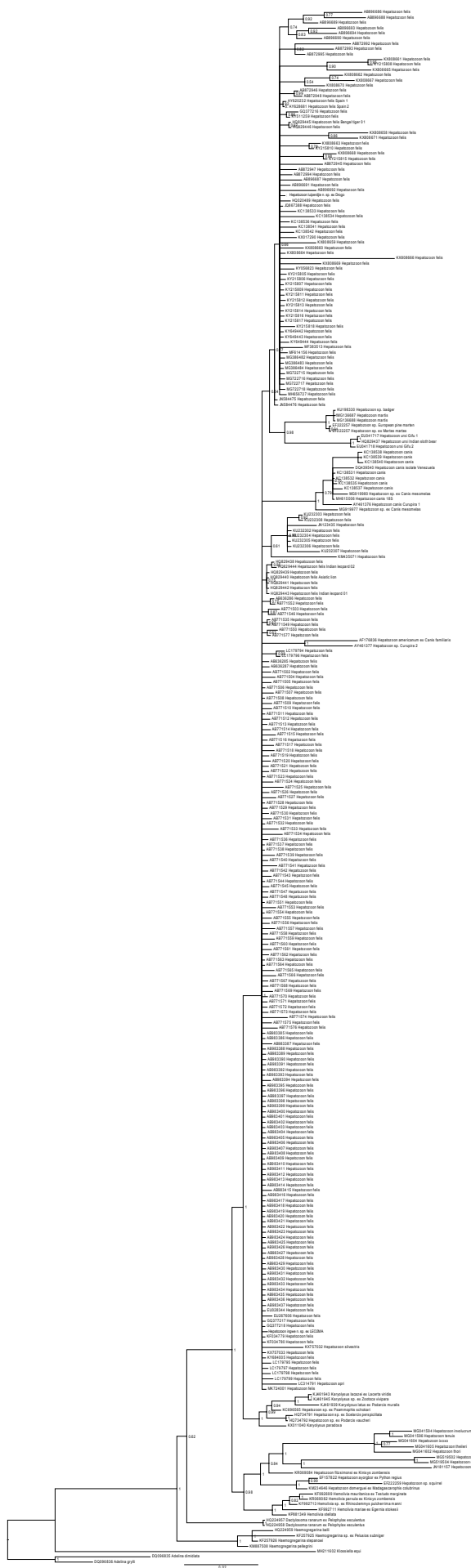

Supplement: Supplementary file 1 — Additional file 1: Figure S1. Bayesian inference (BI) phylogram based on 297 18S rDNA sequences illustrating the phylogenetic relationships between H. felis, H. luiperdjie n. sp. and H. ingwe n. sp. (shown in bold) and other species of Dactylosoma, Haemogregarina, Hepatozoon, Karyolysus and Hemolivia retrieved from GenBank. Adelina dimidiate, A. grylli and Klossiella equi were selected as the outgroup. The scale-bar represents 0.02 nucleotide substitutions per site. [file 13071_2020_3933_MOESM1_ESM.pdf]
